# Acute Hepatitis Secondary to the Use of *Ilex paraguariensis* (Mate Tea): A Case Report and Review of Literature

**DOI:** 10.1155/2019/8459205

**Published:** 2019-10-13

**Authors:** Eduardo A. Rodriguez, Raquel Teixeira Yokoda, David E. Payton, Rish Pai, Thomas J. Byrne

**Affiliations:** Mayo Clinic Arizona, 5777 E Mayo Blvd, Phoenix, AZ 85054, USA

## Abstract

Drug induced liver injury is a very frequent cause of hepatotoxicity and within that group, herbal and dietary supplements are a well described subcategory. The following clinical vignette describes the case of a young man with acute hepatitis secondary to the use of *Ilex paraguariensis*, also known as yerba mate, which is a herbal product commonly drunk in South America. This is the first written case of mate tea induced hepatotoxicity.

## 1. Introduction

Drug induced liver injury (DILI) can occur following the use of both prescription and/or over the counter medications. Even though it is not as frequent as other more common causes of liver injury such as viral hepatitis, it remains to be an important differential diagnosis category that the smart clinician should always consider. Within this category herbal and dietary supplements (HDS) are a frequent cause of hepatotoxicity [1]. The following clinical vignette describes the case of acute hepatitis secondary to the use of *Ilex paraguariensis*, also known as yerba mate.

## 2. Case Presentation

A 21-year-old American gentleman without significant past medical history was transferred from northern Argentina for further investigation of elevated liver enzymes. He had spent the last four months over there as part of his church mission and during the latter two weeks of vacation had initially noted mildly icteric sclera, followed by generalized malaise, jaundice, pruritus, and dark urine. He denied fever, chills, diaphoresis, alcohol intake, over the counter medications, illicit drug use including IV drugs, and recent sexual encounters. He also denied other abdominal complaints such as pain, bloating, nausea, vomiting, and diarrhea. Initial workup abroad included negative viral hepatitis serologies, negative autoimmune markers as well as negative magnetic resonance cholangiopancreatography. Due to the lack of improvement, the family of the patient decided to return him to the U.S. for further evaluation.

On presentation his vital signs were stable. His physical exam was normal except for jaundice. He did not have any neurological deficit. The laboratory findings were as follows: total bilirubin, 32.9 mg/dL: direct bilirubin > 18.0 mg/dL, alanine aminotransferase (ALT), 2685 U/L; aspartate aminotransferase (AST), 1842 U/L, alkaline phosphatase (ALP) 129 U/L. RUCAM score was 65.4. He had negative viral hepatitis markers including hepatitis A, B, C, E, cytomegalovirus, herpes simplex, adenovirus, and varicella zoster virus.

Autoimmune panel including anti smooth muscle antibody (ASMA), anti nuclear antibody (ANA), anti mitochondrial antibody (AMA) and LKM1 antibody were all negative/unremarkable. Further serologies for *Entamoeba histolitica* and *Schistosoma mansoni* were also obtained given the geographical location of his mission, and these were negative. Urine toxicology screen was negative for alcohol, amphetamines, barbiturates, benzodiazepenes, cocaine, opiates, and tetrahydrocannabinol. Acetaminophen level in blood was undetectable (Table 1).

Upon further questioning he reported daily yerba mate tea during the four months he spent in Argentina, sometimes twice a day and symptoms began during the last two weeks he stayed there. He continued drinking the tea until the last day of his vacations, before coming back to the United States. He also added the fact that all of his co-workers had drunk the same tea on a daily basis however no one else developed similar complaints.

**Figure 1:**
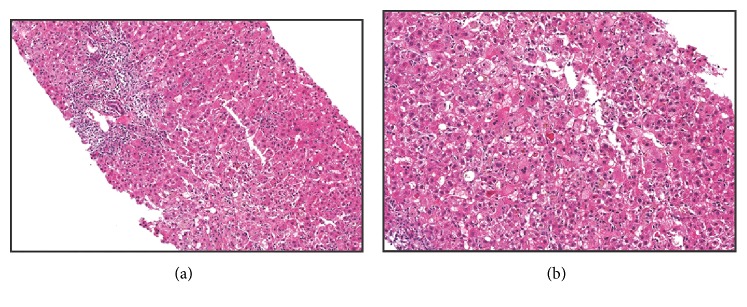
Acute cholestatic hepatitis. (a) Portal inflammation with bile ductular reaction and periportal hepatocyte injury (H&E 100X). (b) Lobular disarray characterized by lobular inflammation, acidophil bodies, and cholestasis (H&E 200X).

In order to further investigate the etiology of his acute hepatitis an ultrasound-guided liver biopsy was obtained. Histological evaluation revealed an acute cholestatic hepatitis pattern, without typical features for autoimmune hepatitis. It demonstrated expanded portal tracts with a mixed inflammatory cell infiltrate composed of lymphocytes as well as occasional eosinophils and neutrophils. A mild bile ductular reaction was also present at the periphery of these portal areas, likely in response to the hepatocellular injury and likely explaining the elevated RUCAM score. Significant lobular disarray with numerous foci of lobular inflammation and acidophil bodies was appreciated. Iron stain demonstrated scattered Kupffer cell iron. PAS with diastase stain was negative for intracytoplasmic globules. Plasma cells were not prominent. The trichrome and reticulin stains confirmed the absence of fibrosis. Overall, the morphologic changes noted were thought to be found most commonly in the setting of medication or toxin-induced injury (including herbal medication) (Figure 1).

**Figure 2:**
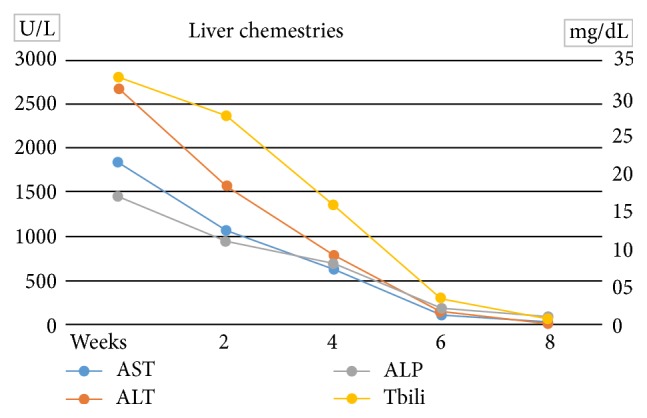
Trend of liver enzymes during admission and in the outpatient setting.

Even though liver markers initially rose, after two days hepatic panel numbers started to downtrend, and the patient was discharged from the hospital. In the outpatient setting patient was monitored closely with liver panel labs. After two months of close follow up, all numbers came back to normal levels, and patient was discharged from clinic (Figure 2). Except for the temporary jaundice and malaise, he remained asymptomatic throughout.

## 3. Discussion

HDS liver injury shares the same underlying biochemical process with DILI in which the foreign chemical needs to be metabolized in order to be eliminated. It is during that process that potential hepatotoxic metabolites can be produced and cause injury in susceptible patients [2]. Most cases of herbal hepatotoxicity reflect an idiosyncratic pattern, which means reactions can occur unpredictably in the population. The other group includes those products that cause intrinsic injury, which means predictable reactions in humans or in animal models when enough dose of the offending agent is administered. Acetaminophen is the prototypic cause of intrinsic injury [1]. In order to consider DILI, other more common etiologies should be rule ruled out first. In this case, we performed a thorough evaluation including viral hepatitis panel as well as autoimmune markers due to the significantly elevated transaminases initially noted, and given the young age of the patient.

After an extensive evaluation which included a liver biopsy that ultimately suggested medication/toxin (including possible HDS) effect, we consider that the main cause of the acute hepatitis in this case was *Ilex paraguariensis, *also known as yerba mate, which the patient drank while in Argentina. The present case describes the first reported case of HDS injury secondary to the use of yerba mate, a common herbal product that is drunk as a tea in the southern portion of South America, namely Argentina, Brazil, Paraguay, and Uruguay [3]. In these countries the leaves and stems of the plant are processed in the production of several types of beverages including mate or chimarrao (warm), as well as various types of teas and carbonated drinks. Some of the attributed biological properties include an antioxidant and hypocholesterolemic capacity as well as possible cancer prevention properties [4]. It is also possible that some of these drinks might have the presence of adulterants that may be incorporated into the final product, either intentionally or unintentionally.

Pathogenesis is difficult to characterize in HDS injury as this is mainly a human and not animal process and thus experimental data on animals are limited. However, some of the available experimental studies have described an intrinsic pattern of HDS with the possible involvement of unsaturated pyrrolizidine alkaloids (PAs). These can damage the endothelial cells of the liver and reduce the sinusoidal blood flow, causing clinical features of hepatic sinusoidal obstruction syndrome [2]. In our case, the hepatotoxic pattern was cholestatic which correlates with a possible hepatic sinusoidal obstruction syndrome-like presentation.

Liver injury from HDS is a growing and challenging problem. Further clinical and basic science research as well as better monitoring and regulatory efforts is needed in order to insure consumer safety [5].

## Figures and Tables

**Table 1:** Laboratory data on admission.

*Complete blood count*		
White blood cell count	5900	/*µ*L
Hemoglobin	14.2	g/dL
Platelets	350	10^3^/*µL*

*Coagulation*		
PT	13.6	sec
INR	1.2	

*Complete metabolic panel*		
Albumin	4.7	g/dL
Total bilirubin	32.9	mg/dL
Direct bilirubin	>18	mg/dL
AST	1842	U/L
ALT	2685	U/L
ALP	129	U/L
Lipase	20	U/L
BUN	7.5	mg/dL
Creatinine	0.69	mg/dL
eGFR	>90	mL/min

*Immunology*		
ANA	0.4	Dil
AMA	<0.1	Index
anti-LKM1 Ab	<5.0	Index
ASMA	Negative	Index
Alpha-antitrypsin	177	mg/dL

*Infectious markers*		
HCVAb	Negative	
HCV-RNA	Undetectable	
HBsAg	Negative	
HBcgM Ab	Negative	
HAV-IgM	Negative	
HEV-IgM	Negative	
HTLV-1 Ab	Negative	
EBV-IgG	Negative	
EBV-IgM	Negative	
CMV-IgG	Negative	
CMV-IgM	Negative	
VZV-IgG	Negative	
VZV-IgM	Negative	
HSV-IgG	Negative	
HSV-IgM	Negative	
Adenovirus PCR	Negative	

*Toxicology and drug testing*		
Acetominophen, S	<5.0	mcg/mL
Ethanol, U	Undetectable	
Amphetamines, U	Undetectable	
Barbiturates, U	Undetectable	
Benzodiazepines, U	Undetectable	
Cocaine, U	Undetectable	
Methadone metabolite, U	Undetectable	
Opiates, U	Undetectable	
Tetrahydrocannabinnol, U	Undetectable	
